# Epidemiologie der Skabies in Deutschland: Multi-Source-Analyse von Primär- und Sekundärdaten

**DOI:** 10.1007/s00105-021-04895-1

**Published:** 2021-10-04

**Authors:** Matthias Augustin, Claudia Garbe, Gefion Girbig, Klaus Strömer, Natalia Kirsten

**Affiliations:** 1 Institut für Versorgungsforschung in der Dermatologie und bei Pflegeberufen (IVDP), Universitätsklinikum Hamburg-Eppendorf (UKE), Martinistr. 52, 20246 Hamburg, Deutschland; 2 Berufsverband der Deutschen Dermatologen e. V. (BVDD), Robert-Koch-Platz 7, 10115 Berlin, Deutschland

**Keywords:** Hautkrankheiten, Versorgungsforschung, Krankenkassen, Häufigkeit, Multi-Source-Analyse, Skin diseases, Health services research, Sickness funds, Frequency, Multi-source analysis

## Abstract

**Hintergrund:**

Skabies stellt weltweit eine der häufigsten und hinsichtlich der Krankheitslast bedeutendsten Hautkrankheiten dar. In Deutschland wird derzeit eine Zunahme von Fällen diskutiert, wofür bisher belastbare Zahlen fehlten.

**Fragestellung:**

Häufigkeit und Versorgungsmerkmale der Skabies in Deutschland.

**Material und Methode:**

Multi-Source-Analysen aus Versorgungsdaten einer bundesweiten gesetzlichen Krankenversicherung, des Statistischen Bundesamtes und von betrieblichen Hautscreenings.

**Ergebnisse:**

In Deutschland weist Skabies seit 2009 und insbesondere seit 2014 eine steigende Versorgungsprävalenz auf. Im ambulanten Bereich findet sich ein Anstieg zwischen 2010 und 2015 von 52,8 % auf etwa 128.000 Behandlungsfälle. Stationär werden in Deutschland derzeit jährlich über 11.000 Fälle mit Skabies als Hauptdiagnose (ICD-10 B86) dokumentiert. Der Anstieg zwischen 2010 und 2016 betrug etwa 306 %. Hauptversorgende ambulante Fachgruppen sind Dermatologen und Hausärzte, im stationären Bereich Fachabteilungen für Dermatologie, Pädiatrie und Innere Medizin.

**Schlussfolgerung:**

Der Versorgungsbedarf wird zukünftig aufgrund der vorgenannten Entwicklung von Prävalenz und Inzidenz weiter auf einem hohen Niveau bleiben, was einen erhöhten Aufklärungs- und Früherkennungsbedarf nahelegt.

## Abstract (en)

**Background:**

Scabies is one of the most common and, in terms of burden of disease, one of the most significant skin diseases worldwide. In Germany, an increase in cases is currently being discussed, for which reliable data have been lacking until now.

**Objectives:**

The goal is to clarify the prevalence and treatment of scabies in Germany.

**Materials and methods:**

Multisource analyses of treatment data from a nationwide statutory health insurance company, the Federal Statistical Office and company skin screenings.

**Results:**

In Germany, the number of cases of scabies has been rising since 2009 and especially since 2014. In the outpatient setting, there was an increase of 52.8% to around 128,000 treatment cases between 2010 and 2015. Currently, more than 11,000 inpatient cases are documented annually in Germany with scabies as the main diagnosis (ICD-10 B86). The increase between 2010 and 2016 was about 306%. The main outpatient specialist groups providing care are dermatologists and general practitioners, while in the inpatient sector treatment is provided by departments of dermatology, paediatrics and internal medicine.

**Conclusion:**

Due to the aforementioned development of prevalence and incidence, the need for care will remain at a high level in the future, which suggests an increased need for education and early detection.

## Hintergrund

Skabies stellt weltweit eine der häufigsten und in der globalen Krankheitslast eine der bedeutendsten Hautkrankheiten dar [4, 8, 10]. Obwohl Skabies grundsätzlich nicht lebensbedrohlich ist, geht von ihr meist ein hoher Leidensdruck aus [16]. Auch kann besonders in ärmeren Regionen ein erhebliches Komplikationspotenzial vorliegen [2, 6], sodass weltweit eine internationale Allianz gegen Skabies gegründet wurde [5]. In der Regel kann Skabies sachgerecht klinisch diagnostiziert [9, 15] und mit den empfohlenen Therapeutika wirksam behandelt werden [3]. Zur Sicherstellung einer leitliniengerechten Versorgung wurde in Deutschland eine AWMF(Arbeitsgemeinschaft der Wissenschaftlichen Medizinischen Fachgesellschaften)-Leitlinie konsentiert [14].

Wenngleich Skabies in Deutschland im internationalen Vergleich weniger häufig zu sein scheint und gut beherrschbar ist, wurde in letzter Zeit ein vermehrtes Aufkommen diskutiert [13]. Zur Versorgungsplanung fehlen allerdings bisher systematische, aktuelle Daten.

Die vorliegende Studie hat die Zielsetzung, sowohl die Epidemiologie wie auch die Versorgung der Skabies als Grundlage für die zukünftige Planung abzuleiten.

## Fragestellungen

1. Wie häufig ist Skabies derzeit in Deutschland?
2. Wie häufig wird Skabies ambulant, wie häufig stationär versorgt?
3. Durch welche Fachabteilungen/-gruppen findet diese Versorgung statt?
4. Wie hoch ist der aktuelle und zukünftige Versorgungsbedarf für Skabies?

## Methoden

### Studiendesign

Durchgeführt wurde eine versorgungswissenschaftliche Analyse auf der Basis von Sekundärdaten [12] mit deskriptivem Ansatz. Eingesetzt wurden Analyseverfahren der klinischen Epidemiologie, Gesundheitsökonomie und Sozialwissenschaften. Sachstand der Analyse ist Juli 2019, die aktuellsten Daten stammen aus den Jahren 2015 (GKV-Daten) und 2017 (stationäre Behandlung).

Zielindikation war Skabies, welche in Deutschland durch die ICD-10-Kodierung B86 (ICD-10-GM) dokumentiert wird [7]. Hinzuweisen ist allerdings auf fehlende klinische Differenzierungen, die mit dem ICD-10-Schlüssel nicht abbildbar sind. Auch beruhen die Daten auf nicht verifizierten Diagnosestellungen.

### Epidemiologie der Skabies – Primärdatenanalyse

Die Daten zur Epidemiologie in Deutschland wurden nicht nur auf der Basis von Sekundärdaten, sondern auch in einer populationsbezogenen Studie erhoben. In Letzterer wurden bundesweit über 200.000 Personen im erwerbsfähigen Alter in über 500 Unternehmen verschiedener Branchen mittels strukturierter Anamnese sowie klinischem Ganzkörperbefund untersucht [1]. Skabiesfälle wurden hier nicht als Fokus, sondern nur vom Untersuchenden bei Vorliegen in Freitexten erfasst.

### Häufigkeit der stationären Fälle

Die Häufigkeit der stationären Fälle wurde aus den bundesweiten Krankenhausdaten des Statistischen Bundesamtes (Destatis-Daten) gewonnen. Die Statistik stellt eine Vollerfassung der stationären Versorgung dar. Die Analyse erfolgte deskriptiv für die Jahre 2000 bis 2017 unter Verwendung des ICD-10-Codes B86 a) für die gesamte Diagnosegruppe, b) nach Fachabteilung und c) für beide geschlüsselt nach Geschlecht. Erfasst wurde dabei lediglich dieser Code als Hauptdiagnose.

Eine Prüfung der Ergebnisse und die Nutzung der Daten für weitere Auswertungen erfolgten aus dem Auswertungssatz einer gesetzlichen Krankenversicherung (DAK-Gesundheit) mit ca. 5,9 Mio. Versicherten, deren Daten bereits in Vorstudien eingesetzt wurden [11]. Im Zuge einer Projektanalyse zu chronischen Entzündungskrankheiten der Haut wurde eine Stichprobe von 40 % der Daten entnommen.

### Anteil der Fachabteilungen

Als Fachabteilungsschlüssel wurde in der Destatis-Krankenhausstatistik der vom Destatis eingesetzte Schlüssel verwendet. Die Krankenhausversorgung wurde ausschließlich auf Fallebene analysiert, da diese auch Abrechnungs- und Vergleichsgrundlage ist. In gleicher Weise wurden die Fachabteilungsschlüssel für die Analyse der Sekundärdaten herangezogen.

### Ambulante Versorgung

Die Leistungen in der ambulanten Versorgung wurden anhand der GKV-Daten analysiert. Grundlage waren hier Fallziehungen aus einer 40 %-Stichprobe der DAK-Gesundheit für die Jahre 2010 bis 2015. Als Kodierung wurde wiederum der ICD-10-Code B86 verwendet. Zur Vermeidung einer Unterschätzung der Fallzahl im ambulanten Bereich wurden angesichts der Akuität von Skabies auch Diagnosen in nur einem Quartal erfasst.

### Analyse des aktuellen und zukünftigen Versorgungsbedarfes

Der aktuelle und der zukünftige Versorgungsbedarf wurden auf der Grundlage der Epidemiologie von Skabies, der daraus festgestellten Behandlungsbedarfe sowie der zukünftigen Epidemiologie im Zuge der demografischen Entwicklung ermittelt.

## Ergebnisse

### Ambulante Versorgung

**Figure 1:**
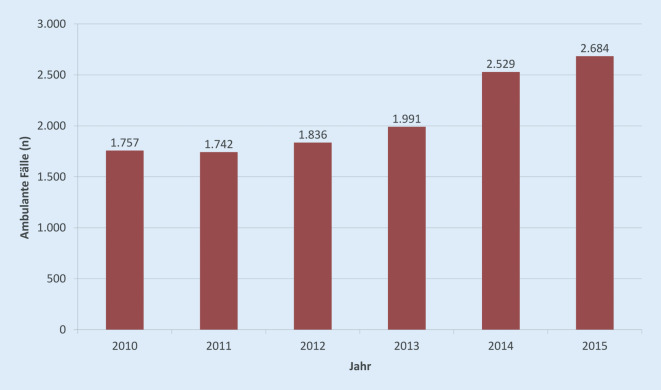

Die Leistungen in der ambulanten Versorgung wurden anhand der GKV-Daten analysiert. Insgesamt wurden im Jahr 2015 innerhalb der Stichprobe von 2,5 Mio. DAK-Versicherten *n* = 2684 Fälle (0,107 %) verzeichnet (Abb. 1) gegenüber *n* = 1757 Fällen in 2010. Damit lag der Anstieg an Behandlungen zwischen 2010 und 2015 bei 52,8 %.

Aus diesen Daten resultiert eine hochgerechnete nicht-adjustierte Prävalenz der ambulant behandelten Skabies in Deutschland für das Jahr 2015 von etwa 0,107 %, entsprechend 88.000 Fällen.

### Häufigkeit der stationären Fälle von Skabies

**Figure 2:**
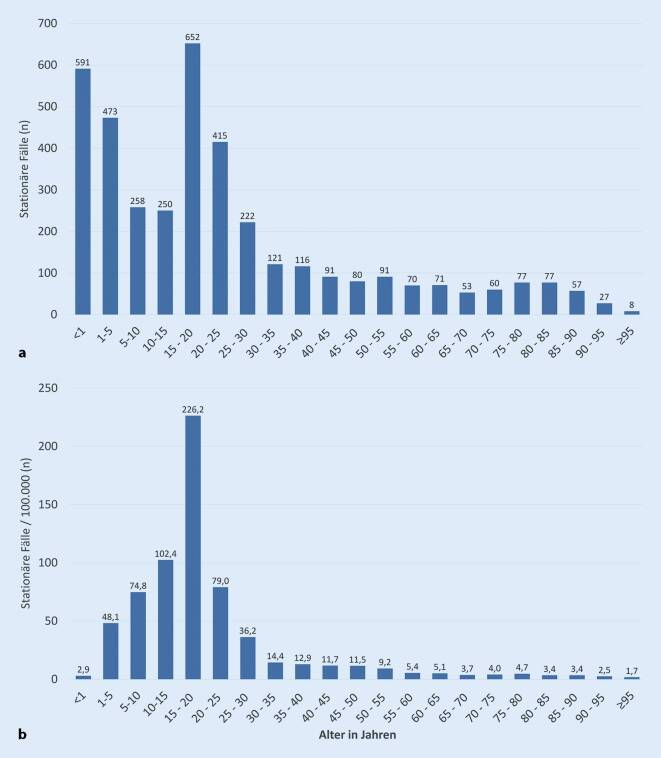

Im Jahr 2016 wurden von Destatis *n* = 3860 stationäre Fälle der Diagnose Skabies/ICD-10 B86 dokumentiert (Abb. 2). Betroffen waren alle Altersgruppen, wobei ein Schwerpunkt bei Kindern und jungen Erwachsenen bis 25 Jahre zu erkennen ist (Abb. 2a). Bezogen auf 100.000 stationäre Fälle pro Altersgruppen lag der Anteil der Skabies im Alter von 15 bis 20 Jahren mit über 200/100.000 am höchsten (Abb. 2b).

Für 2017 liegen in der Berichterstattung von Destatis zum Zeitpunkt der Analyse nur die stationären Behandlungstage vor, nicht aber die Fälle. Von diesen Fällen betrafen 51 % Männer und 49 % Frauen, die insgesamt 21.976 stationäre Behandlungstage induzierten (Tab. 1).

**Table 1:** 

Fachabteilung	Männer (*n*)	Frauen (*n*)	Gesamt (*n*)	Anteil weiblich (%)	Anteil Fachgruppe (%)
Dermatologie	8386	7772	16.158	48,1	73,5
Pädiatrie	2305	2491	4796	51,9	21,8
Innere Medizin	376	228	604	37,7	2,7
Andere	233	185	418	44,3	1,9
Gesamt	11.300	10.676	21.976	48,6	100,0

### Verlauf der stationären Fälle 2000 bis 2017

**Figure 3:**
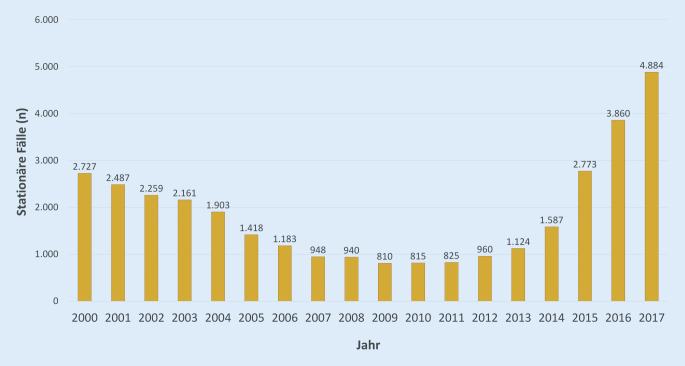

Im Verlauf von 2000 bis 2017 zeigt sich ein U‑förmiger Verlauf: Während in den Jahren 2000 bis 2006 noch über 1000 Fälle als stationäre Hauptdiagnosen dokumentiert wurden, sank dieser Anteil nachfolgend bis auf 810 im Jahr 2009 (Abb. 3). Seit dem Jahr 2014 ist wieder ein markanter, bisher anhaltender Anstieg zu verzeichnen.

Im Vergleich der Jahre 2009 bis 2017 lässt sich insgesamt ein Anstieg der stationären Fälle von 810 um 376,5 % auf 5816 feststellen (Tab. 2). Im Vergleich dazu stieg die Anzahl stationärer Krankenhausfälle in Deutschland nur um 9,4 %, die der stationären dermatologischen Diagnosen (L00-L99) um 20,2 %.

**Table 2:** 

	2009	2017	Veränderung (%)
Stationäre Fälle Skabies (*n*)	810	5816	618,0
Alle stationären Fälle in Deutschland (*n*)	18.231.569	19.952.735	9,4
Anteil Skabies pro 100.000 Krankenhausfälle	4,4	29,1	556,1
Alle dermatologischen Diagnosen (*n*)	252.203	303.272	20,2
Stationäre Behandlungen wegen Skabies in Relation zu stationären dermatologischen Diagnosen (L00-L99) (%)	0,3	1,9	497,1

Dementsprechend stieg der Anteil der Skabiesfälle an den stationären Krankenhausfällen insgesamt um über 500 %, die Zahl der Skabiesfälle in Relation zur Zahl der stationären Hautdiagnosen stiegt ebenfalls überproportional um 497 %.

Geht man von den ermittelten 127.952 ambulanten Fällen und 3860 stationären Fällen von Skabies in 2015 aus, so wurde ein Anteil von 3,0 % der ambulanten Fälle auch stationär behandelt. Dieser Quotient lag 2010 noch bei 1,3 % (815 stationäre Fälle auf 61.723 ambulante).

### Anteil der Fachabteilungen

Hauptversorgende Abteilungen in 2017 (Tab. 1) waren die Dermatologie (73,5 % Anteil), gefolgt von der Pädiatrie (21,8 %) und der Allgemeinen Inneren Medizin (2,7 %). Lediglich 1,9 % wurden durch andere Fachabteilungen versorgt.

### Primärdatenanalyse

Das Durchschnittsalter der 200.000 untersuchten Personen in den Betrieben betrug 46,1 ± 9,8 Jahre, 53,5 % waren männlich, und der mittlere Body-Mass-Index (BMI) betrug 26,1 ± 4,4 kg/m^2^ [1]. Erwartungsgemäß wurde hier praktisch keine Skabies festgestellt. Grund hierfür sind a) der Healthy-Worker-Effekt, b) damit verbunden die fehlende Risikolage der meisten Beschäftigten für Skabies und c) die Akuität der Erkrankung, welche im Eintretensfall schnell zu versorgen und auszukurieren ist. Der sporadische Charakter der Erkrankung trägt auch allgemein zu einer geringen Verbreitung in Betrieben bei.

## Diskussion

Ziel der vorliegenden Analyse war die Ermittlung der Prävalenz von Skabies in Deutschland und daraus die Ableitung des aktuellen und des zukünftigen Versorgungsbedarfes. Sowohl die ambulanten wie auch die stationären Behandlungsdaten lassen eine klare Tendenz zu einer erhöhten Skabiesprävalenz seit 2010 erkennen. Besonders stark ist der Anstieg seit 2014. Daraus ergibt sich unmittelbar ein ebenfalls gestiegener, genereller Versorgungsbedarf. Diese Daten bestätigen Annahmen der AWMF-Leitlinie [14] und der Sekundärliteratur [13] sowie Berichte in den Medien, die bislang jedoch nicht wissenschaftlich hinterlegt waren.

Der besonders stark gestiegene Anteil stationärer Behandlungen bedarf einer gesonderten Betrachtung. Der zwischen 2000 und 2006 beobachtete Abfall dieser stationären Fälle war vermutlich durch die Wandlung des stationären Versorgungssystems im Zuge der G‑DRG(German Diagnosis Related Groups)-Einführung und der Verlagerung von Behandlungen in den ambulanten Bereich bedingt. Angesichts der seitdem nicht mehr veränderten stationären Vergütungsstruktur lassen sich die erneuten Anstiege stationärer Fälle in den letzten Jahren nicht mit entsprechenden Anreizen, sondern einem tatsächlichen Mehrbedarf erklären. Da ein Großteil der Skabieserkrankungen bei rechtzeitiger Diagnosestellung ambulant beherrschbar ist, könnte der Anstieg an zu spät erkannten oder aufgrund sozialer Umstände nicht hinreichend kontrollierbaren Fällen liegen. Inwieweit auch therapierefraktäre Verläufe, Resistenzen oder eine unzureichende diagnostische und therapeutische Versorgungsqualität zu dem Anstieg der stationären Fälle beigetragen haben, bedarf weitergehender, versorgungswissenschaftlicher Analysen.

Eine Limitation der Nutzung von Sekundärdaten aus den gesetzlichen Krankenversicherungen und der Berichterstattung des Bundes ist die fehlende Prüfbarkeit der Diagnosen, die weit überwiegend klinisch gestellt worden sein dürften. Dieser Umstand gilt jedoch für den gesamten Beobachtungszeitraum in gleicher Weise, sodass wir hier keine systematische Verzerrung erwarten.

Die vorliegenden Daten unterstreichen, dass die Skabies zukünftig auch in Deutschland eine stärkere klinische und versorgungswissenschaftliche Forschung benötigt. Hierzu zählen neben der Epidemiologie auch die Resistenzforschung an den zugelassenen Arzneimitteln sowie die Entwicklung neuer Wirkstoffe. In der medizinischen Versorgung ist anzustreben, die Skabies noch stärker in die Wahrnehmung der Ärzteschaft und der Sozialeinrichtungen zu bringen. Sowohl in der ambulanten wie auch in der stationären Dermatologie gilt es, die Versorgung der Skabies durch innovative Versorgungskonzepte und bessere Kooperationen zwischen den Gesundheitsbehörden, den Trägern der Sozialeinrichtungen und der Ärzteschaft weiterzuentwickeln und die Risikogruppen für das massenweise Auftreten frühzeitig zu identifizieren.

## Limitationen

Durch die Datengrundlage ergeben sich folgende Limitationen:

- fehlende Verifizierung der Diagnosen durch Fachärzte für Dermatologie, da es sich um eine Sekundärdatenanalyse von GKV-Daten handelt,
- keine reinen bevölkerungsbezogenen Prävalenzdaten, sondern nur Angaben zur Behandlungsprävalenz,
- Dunkelziffer an Skabiesfällen kann nur mittels Primärdatenanalysen durch aufwendige Populationsstudien ermittelt werden.

## Ausblick

Vorerst ist auf der Basis der vorliegenden Daten davon auszugehen, dass auf allen Ebenen interveniert werden sollte: a) Forcierung der Primär- und Sekundärprävention in Risikogruppen, b) frühzeitigere Entdeckung von Skabiesfällen durch mehr Aufklärung und edukatorische Maßnahmen in den Gesundheitsberufen, c) Tertiärprävention bei erkannten Risikogruppen und d) konsequente Nutzung der therapeutischen Optionen nach Leitlinie.

Aufgrund ihrer deutlich gestiegenen Prävalenz kommt die Skabies nicht nur als Primärdiagnose vor. An sie muss auch in der Differenzialdiagnostik von Dermatosen gedacht werden.

## Fazit für die Praxis

- Aufgrund ihrer deutlich gestiegenen Prävalenz kommt die Skabies nicht nur als Primärdiagnose vor. An sie muss auch in der Differenzialdiagnostik von Dermatosen gedacht werden.
- Das Management der Erkrankung geht über die Dermatotherapie weit hinaus und umfasst insbesondere das Verhaltensmanagement. Für die Praxis ist der Einsatz digitaler Techniken (Online-Informationen, Hinweisvideos) zur Unterstützung des Dermatologen empfehlenswert.
- Aufgrund der nur mäßigen Compliance bei vielen Patienten bieten sich digitale Apps mit Recall-Funktionen zur Gewährleistung der Karenzmaßnahmen an.
